# (Dis-)Harmony in movement: effects of musical dissonance on movement timing and form

**DOI:** 10.1007/s00221-015-4233-9

**Published:** 2015-03-01

**Authors:** Naeem Komeilipoor, Matthew W. M. Rodger, Cathy M. Craig, Paola Cesari

**Affiliations:** 1 Department of Neurological and Movement Sciences, University of Verona, Via Casorati 43, 37131 Verona, Italy; 2 MOVE Research Institute, VU University Amsterdam, 1081 BT Amsterdam, The Netherlands; 3 School of Psychology, Queen’s University Belfast, David Keir Building, 18-30 Malone Road, Belfast, BT9 5BN UK

**Keywords:** Consonance dissonance sounds, Musical pitch intervals, Sensorimotor synchronization

## Abstract

While the origins of consonance and dissonance in terms of acoustics, psychoacoustics and physiology have been debated for centuries, their plausible effects on movement synchronization have largely been ignored. The present study aimed to address this by investigating whether, and if so how, consonant/dissonant pitch intervals affect the spatiotemporal properties of regular reciprocal aiming movements. We compared movements synchronized either to consonant or to dissonant sounds and showed that they were differentially influenced by the degree of consonance of the sound presented. Interestingly, the difference was present after the sound stimulus was removed. In this case, the performance measured after consonant sound exposure was found to be more stable and accurate, with a higher percentage of information/movement coupling (tau coupling) and a higher degree of movement circularity when compared to performance measured after the exposure to dissonant sounds. We infer that the neural resonance representing consonant tones leads to finer perception/action coupling which in turn may help explain the prevailing preference for these types of tones.

## Introduction

We interact with our environment through movement; with the way we move being influenced by many different types of perceptual information. For instance, environmental sounds carry an ecological significance that allows us to move in the direction of an object, detect the presence of objects, interact with others and even interpret events using sound alone (Gaver 1993; Carello et al. 2005). One of the key ways in which humans naturally interact with their auditory environment is when they synchronize their movements to regular patterns of sound (e.g., dancing to a beat). Indeed, to be able to synchronize movements to sounds, an activity humans are very skilled at, the nervous system must pick up information from the auditory perceptual stream about the time until the next beat sounds and use this information to prospectively guide the generation of consecutive actions (Craig et al. 2005). Given the absence of a continuous source of external temporal information to guide the action extrinsically, the nervous system must create its own source of dynamic temporal information (Tau-G, Craig et al. 2005). It has already been shown that the structure of sound events (discrete vs. continuous) can affect the processes by which movements are timed to sounds, even if the interval durations are the same (Rodger and Craig 2011, 2013). Although synchronization of body movement to the perceived musical tempo has been widely studied (see Repp and Su 2013, for a review), the effects of other aspects of auditory stimuli on movement–sound synchronization, such as musical pitch relationships, have largely been neglected.

Synchronizing movements with musical rhythms is indeed one of the most natural and instinctive ways in which humans interact with their auditory environment. The inextricable link between sound and movement forms the basis of music and dance performance. Interestingly, it has been shown that music and movements share similar structures and present common cross-cultural expressive codes (Sievers et al. 2013). In the same vein, the evaluation of the emotional content of observed biological motion (point-light displays of human motion) has been shown to be strongly influenced by the presence of accompanying music (Kaiser and Keller 2011). Already from the first month of life, infants move their body more naturally under the presence of musical rhythm than speech rhythm (Zentner and Eerola 2010), being able not only to synchronize correctly their movements with the different musical tempo but also being selectively sensitive to melodies presenting different pitch structures (Zentner and Kagan 1998). In a different scenario, human adults have been shown to use a different walking strategy under the guidance of music than under a metronome beat (Styns et al. 2007; Wittwer et al. 2013). A number of studies have revealed that musical rhythm can even enhance motor performance in Parkinson’s disease (PD) (Thaut and Abiru 2010; Satoh and Kuzuhara 2008; Lim et al. 2005). Moreover, using a finger-tapping paradigm, it has been shown that synchronization error was significantly less when tapping with music cues than metronome ones (Thaut 1997). What emerges from these studies is that in addition to the timing cues music conveys, other properties also help guide the coordination of movement. Hence, investigating whether and how non-temporal cues, such as pitch and harmony, influence movement synchronization is crucial for understanding the inseparable connection between action and perception.

Consonant and dissonant pitch relationships in music provide the basis of melody and harmony. It has been recognized since antiquity that musical chords are either consonant (sounding pleasant or stable) or dissonant (sounding unpleasant or instable). Although composers make use of both intervals to evoke diverse feelings of “tension” and “resolution,” consonant intervals in tonal music occur more often than the dissonant ones (Vos and Troost 1989). Consonant intervals are more preferred also by human infants (Trainor et al. 2002; Zentner and Kagan 1998; Masataka 2006). Remarkably, the preference of consonance over dissonance seems to be cross-cultural, as it has been reported among native African populations who did not have prior experience with Western music (Fritz et al. 2009). Moreover, Schwartz et al. (2003) found a correlation between musical consonance rankings and the probability distribution of amplitude–frequency of human utterances, suggesting that the preference for musical pitch intervals is based on similar physical principals that rule human vocalization (Schwartz et al. 2003). Overall, it seems that some characteristics of musical pitch interval perception might be innate and represent a by-product of fundamental biological properties.

While we can identify differences in the preference and occurrence of consonant and dissonant pitch intervals in nature, it is also possible to define these differences at a mathematical or physical level. The Greek scholar Pythagoras defined the occurrence of consonance as being when the length of string segments forms simple integer ratios (e.g., 3:2, 2:1) with dissonant intervals being when string length ratios are more complex (e.g., 16:15, 243:128). Hermann von Helmholtz argued that consonance occurs not only as a consequence of simple frequency ratio relationships, but also as a result of the interference between overtones of slightly different frequencies—a phenomenon known as *beating*. When the harmonics of complex tones are close, the beating gets faster and forms an unpleasant sensation called roughness (Helmholtz 1954)

A number of studies have attempted to investigate the neuronal substrates underlying the perception of consonance and dissonance. Functional magnetic resonance imaging (fMRI) has revealed differences in activation in different brain areas such as the cingulate and frontal gyrus, and the premotor cortex while listening to dissonant over consonant chords (Tillmann et al. 2003; Foss et al. 2007; Minati et al. 2009). A recent EEG study provided evidence that consonance and dissonance activate neural regions associated with pleasant and unpleasant emotional states, respectively (Maslennikova et al. 2013). Other studies have investigated the neural correlates of emotional responses to consonant (pleasant) and dissonant (unpleasant) music (for review, see Koelsch et al. 2006; Sammler et al. 2007). Studies of event-related potentials (ERPs) revealed that such modulations in cortical activity were correlated with the hierarchical ordering of musical pitch (i.e., the degree of consonance or dissonance of different tone combinations in a musical scale) (Brattico et al. 2006; Krohn et al. 2007; Itoh et al. 2010). In a recent study, Bidelman and Krishnan (2009) showed that consonant intervals yield more robust and synchronous phase locking of auditory brainstem responses, that is, the mechanism by which the auditory nerves fire at or near the same phase angle of a sound wave. Importantly, this result is in accord with pervious animal studies revealing a correlation between the perceived consonance of musical pitch relationships and the magnitude of phase-locked activity in the primary auditory cortex (Fishman et al. 2001), the auditory nerve (Tramo et al. 2001) and the midbrain (Mckinney et al. 2001). Together, these studies suggest compelling evidence that musical scale pitch hierarchies are preserved at both cortical and subcortical levels, which indicates that the auditory system is tuned in to the biological relevance of consonant versus dissonant sounds. Importantly, Tierney and Kraus (2013) demonstrated that the ability to synchronize to a beat relates to the phase-locking response in the auditory brainstem; less auditory–motor synchronization variability when tapping to a beat is associated with more consistent responses in the auditory brainstem. Hence, a more stable neural representation of consonant intervals compared with dissonant ones could lead to a more stable motor output even during the continuation phase where no external pacing stimulus is present. The latter might happen due to different emotional states evoked by sounds during the synchronization phase, which might last during the continuation phase and in turn affect the types of movements produced.

Given the suggested ecological relevance of consonance/dissonance, it is possible that the harmonic structure of sounds may affect the spatiotemporal characteristics of movements when using such sounds to guide timed actions. Our study addresses this issue in a synchronization–continuation paradigm, in which participants were asked to synchronize their movements with auditory tones and then to maintain the same pattern of movements in the absence of the auditory stimuli. The pair of tones delivered differed in the degree of dissonance (from highly consonant (C & G) to highly dissonant (C & C#). By measuring timing accuracy and variability, along with parameters defining the movement trajectory form, we assessed the effects of auditory consonance/dissonance on participants’ movements.

Finally, we tested the effects of sound on movement by applying a model derived from tau-coupling theory (Craig et al. 2005), which describes how the prospective temporal information generated within the central nervous system (an intrinsic tau-guide) can facilitate the prospective control of movement for synchronizing movement to beats. The intrinsic tau-guide is developed based on general tau theory (Lee 1998), which aims to describe the control of intrinsically paced movements. In terms of sensorimotor synchronization, Craig et al. (2005) postulated that during the synchronization of movement with beats the inter-onset intervals are represented in the form of a “tau-guide,” a dynamic neural representation that prospectively informs individuals about the time remaining to the arrival of the next beat. They reported that individuals accomplish the task by coupling their movement onto the tau-guide where the tau of the movement gap (*τ*_m_—the movement gap divided by its closure rate) is kept in constant ratio to the tau-guide (*τ*_g_—the time-to-sounding of the next beat). Hence, the acoustic information of a metronome’s beat sets the parameters of the intrinsic tau-guide in the nervous system that consequently guides the spatiotemporal unfolding of the synchronization movement. What is not clear yet is whether the structure of an auditory event can differentially affect the tau-coupling procedure and consequently result in different movement timing processes.

Our overall aim was to test whether and how consonant/dissonant pitch intervals affect the spatiotemporal properties of regular reciprocal aiming movements. We hypothesized that (1) both the spatial and temporal dynamics of coordinated movement would differ when synchronizing movement to consonant compared with dissonant tones and (2) such differences in movement will be maintained when the stimuli are removed.

## Methods

### Participants

Thirteen healthy (7 females and 6 males), right-handed adults with no musical training (assessed via a questionnaire) volunteered to participate in the experiment. The mean age was 29.4 years (range 20–38 years).

### Materials and apparatus

**Fig. 1:**
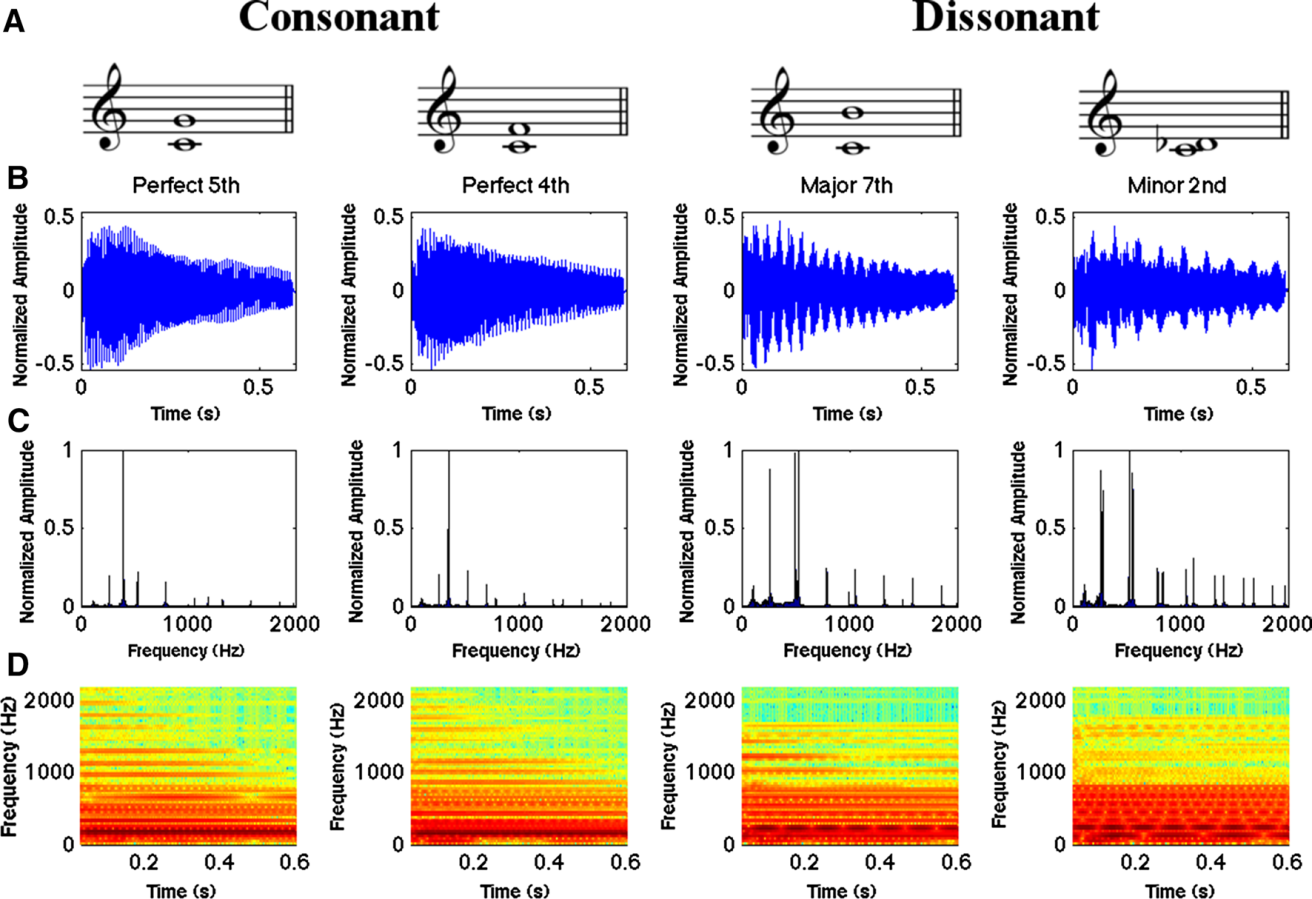
**a** Musical notation. **b** Waveform. **c** Frequency spectra. **d** Spectrograms for the four chords (two consonant and two dissonant) used in the study

A set of four synthesized piano musical dyads (i.e., two-note musical intervals) were constructed as stimuli and presented to participants through noise-isolating headphones at a constant intensity (68 dB SPL). The stimuli consisted of two consonant intervals (perfect fourth: 4:3, perfect fifth: 3:2) and two dissonant intervals (minor second: 16:15, major seventh: 15:8) played back in an isochronous sequence where the inter-onset interval was 0.6 s. Sounds were played for the same duration (0.6 s) with a decreasing amplitude envelope (see Fig. 1b). Sounds were created with Guitar Pro 6 software (www.guitar-pro.com/) (music notation, waveform, frequency spectra and spectrogram for each sound can be seen in Fig. 1). The stimuli were delivered using a Pure Data (http://puredata.info/) patch.

**Fig. 2:**
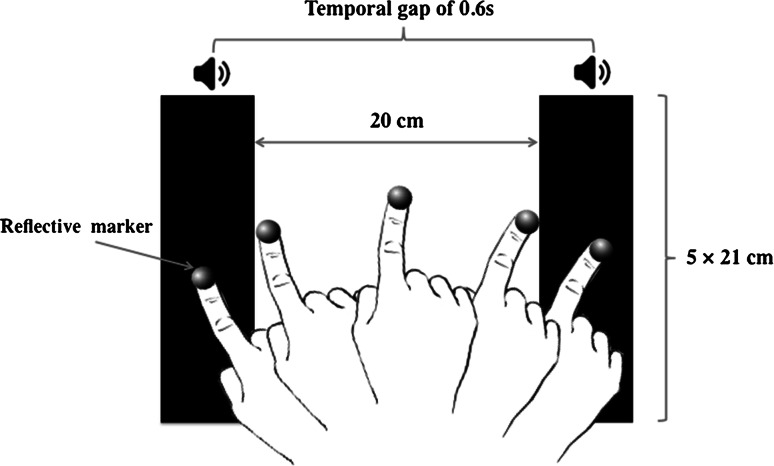
Illustration of the experimental setup where the two *black rectangles* represent the target zones. The duration of the inter-stimulus interval is represented as the temporal gap on the diagram

Participants were asked to sit in front of the table so that the sagittal plane line of their right arm bisected the horizontal plane midway between the two targets. The experimental setup is shown in Fig. 2. Targets were printed on laminated A4 paper. The targets were two 5 × 21 cm black-colored blocks, separated by a white gap of 20 cm. Participants wore a thimble on their right index finger with a mounted reflective marker. Motion data were recorded using 3 Qualisys Oqus 300 Motion Capture cameras connected to a Dell PC running QTM software, sampling at 500 Hz with a spatial accuracy of ±0.1 mm. Before the start of each trial, the coordinates of the target zones were recorded so that the positional data could be calibrated with respect to target position. Motion capture data were synchronized with the sounds presented using the Open Sound Control (http://opensoundcontrol.org/) protocol.

### Procedure

For all trials, participants were given specific instruction to slide their right index fingers between the two target zones in such a way that the stopping of movement in the target zone coincided with the sounding of the metronome beats (synchronization phase). Hence, both the beginning and the end of each movement were defined as the moment when the hand stopped in the target zones. They were also asked to continue moving between the target zones after the metronome had stopped sounding, maintaining the same interval duration between each movement (the continuation phase), until they were instructed to stop moving by the experimenter (see Fig. 2). At the start of each block, participants were presented with 10 repetitions of each sound type so that they could become familiar with the interval duration. Each participant took part in a single session comprised of five blocks of four conditions (four sounds: *perfect fifth, perfect fourth, major seventh* and *minor second*). For each condition, in both the synchronization and the continuation phases, 30 interceptive movements to the targets were recorded (15 to the left side and 15 to the right side). The presentation of the experimental conditions was counterbalanced across participants.

After the synchronization part of the experiment was completed, behavioral valence judgments of consonance and dissonance sounds (pleasantness/unpleasantness) were measured using a rating scale paradigm. The four stimuli used in the experiment (perfect fourth, perfect fifth, major seventh and minor second) were presented to each participant at an intensity of 68 dB through headphones for 4 s. After the presentation of each sound, individuals were asked to rate the valence/pleasantness of each stimulus on a 5-point rating scales where “1” indicated very unpleasant and “5” indicated very pleasant.

### Data analysis

Temporal control of movement was analyzed by examining both the timing and movement trajectory formation (absolute synchronization errors, spread of error, movement harmonicity and tau-guide coupling). Using MATLAB, positional data were filtered using an eight-order low-pass Butterworth filter with a cutoff frequency of 20 Hz (The Mathworks Inc. 2011). The velocity profile was calculated using the first derivative of the smoothed positional data. Synchronization was determined as being the point when the finger stopped moving. The moment representing the end of the finger movement was taken as the first sample that dropped below 5 % of peak velocity for that particular interceptive movement to the target zone. Descriptions of the calculations for each measure are given below.

#### Absolute synchronization errors

Absolute synchronization errors between a participant’s finger movements and the auditory guides were measured for each movement as an absolute difference between the time of auditory stimulus onset and the time when the finger stopped in the target zone. The beats sounded for the same duration as the inter-stimulus interval (0.6 s) with a decreasing amplitude envelope. Synchronization was assumed to be possible, as the beats (chords) had a clear amplitude onset, which has been shown in previous studies to perceptually demarcate the beginning of an auditory event (Phillips et al. 2002).

#### Spread of error

The variability of the synchronization error between consecutive movements for each trial was measured using the spread of error calculation described by Bieńkiewicz et al. (2012). It was measured as the absolute difference between the synchronization errors (with respect to beat onset) made in consecutive movements.

1 $${\text{SpE}} = \frac{{\mathop \sum \nolimits_{i = 1}^{N} |T_{i + 1} - T_{i} |}}{N - 1}$$

where SpE is average spread error, T is temporal error (the difference in time between the onset of the auditory stimulus and the moment the finger stopped in the target zone) and N is the overall number of trials.

#### Movement harmonicity

The harmonicity of the movement (a measure of how sinusoidal the dynamics of individual movements are) was calculated by the formula used in Rodger and Craig (2011). This was calculated by normalizing the absolute velocity profile for each movement so that it fell between 0 and 1 and then interpolating to give 101 data points. The index of circularity was measured by calculating the root mean square error (RMSE) between the normalized velocity–displacement profile and a semicircle, and it was subtracted from 1 (1-RMSE). A semicircle consists of 101 points given by

$$f\left( x \right) = \, 2 \times \surd \left( {x \times \left( {1 - x} \right)} \right),\quad {\text{where }}x \, = \left\{ {0,0.01,0.02, \ldots ,1} \right\}$$

#### Tau-guide coupling

Finally, we tested a model derived from tau-coupling theory (Craig et al. 2005). According to this theory, in order to synchronize movements with auditory beats, one would need to couple the temporal control of movement, or tau of the motion gap *x*, (*τ*_*X*(*t*)_) onto an internal tau-guide that specifies the time-to-sounding of the next beat (*τ*_*g*(*t*)_) at a constant ratio (*k*) so that

2 $$\tau_{X(t)} = k\tau_{g(t)}$$

**Fig. 3:**
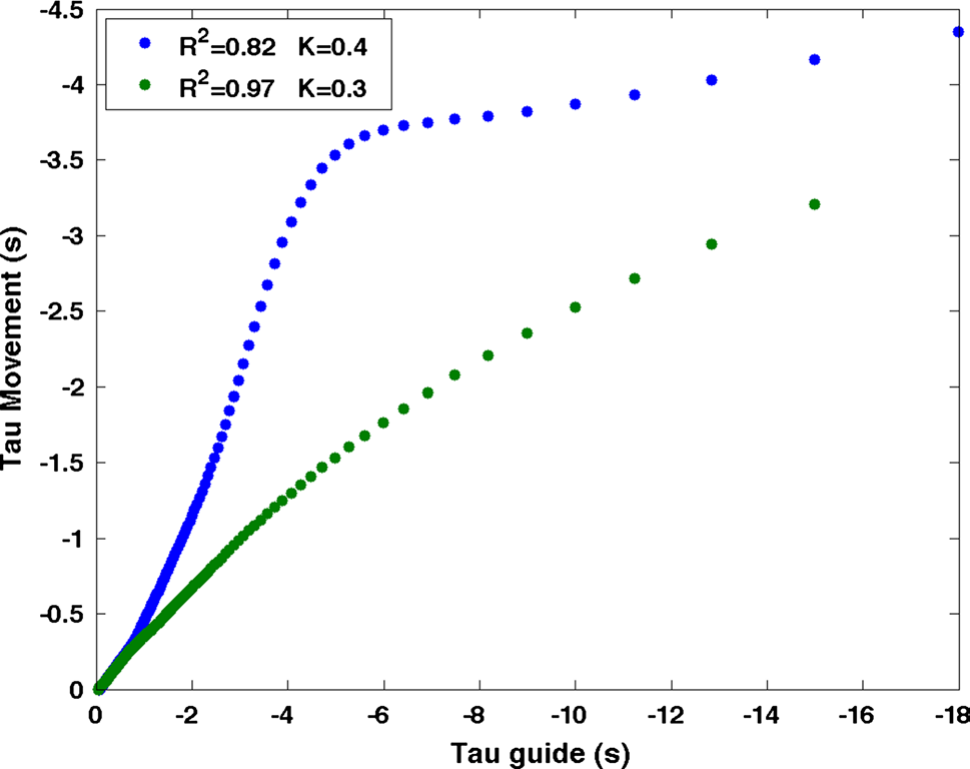
Examples of tau coupling between the tau of the movement gap and the intrinsic tau-guide. The *R* ^2^ values displayed in the top left corner are the linear regression coefficients and *k* values are the coupling constants

The time to closure of a motion gap, tau *x* (*τ*_*X*(*t*)_), specifies the way the movement changes over time and is defined as the ratio between the magnitude of the action displacement gap and its current rate of closure: *X* (displacement)/*Ẋ* (velocity). The intrinsic tau-guide, *τ*_*g*(*t*)_, is derived from Newton’s equations of motion and represents the time to gap closure of a virtual object moving under constant acceleration (Lee 1998),

3 $$\tau_{g(t)} = \frac{1}{2} \times \left(t - \frac{{T^{2} }}{t}\right)$$

with *T* being equal to the inter-beat interval (0.6 s) and *t* the evolving time series within the inter-beat interval. The value *k* is the coupling constant that captures the dynamics of gap closure with different *k* values corresponding to different velocity profiles (Craig et al. 2005). In order to find the strength of coupling, the tau of the movement was linearly regressed against the hypothetical tau-G guide and the strength of the coupling was calculated by the *r*-squared values of the regression analysis, with higher r-squared values indicating a stronger coupling (see Fig. 3).

### Statistical analysis

#### Kinematic data

Two-way repeated-measure ANOVAs [2 sounds (*consonant* and *dissonant*) × 2 *task phase* (*synchronization* and *continuation*)] were carried out on each of the five different variables. Post hoc comparisons were performed by means of *t* tests applying a Bonferroni correction for multiple comparisons when required. A partial eta-squared statistic served as the effect size estimate.

#### Behavioral data

A paired sample *t* test was used to examine the difference between the mean rating of pleasantness for consonant and dissonant sounds. A Cohen’s d statistic was also used as an effect size estimate.

## Results

### Behavioral valence ratings of consonance and dissonance

The average behavioral valence ratings for pleasantness for the four stimuli were found to be higher for the consonant (4.30 ± 0.23 for perfect fifth, 3.61 ± 0.26 for perfect fourth) compared with dissonant sounds (2.69 ± 0.22 for major seventh and 1.92 ± 0.22 for minor second). This ordering of consonance observed here is consistent with previous reports of pleasantness ratings of musical intervals (e.g., Bidelman and Krishnan 2009, 2011; Bidelman and Heinz 2011; Schwartz et al. 2003). A paired *t* test showed that this difference in perceived pleasantness between the consonant sounds and dissonant sounds was significant (*t*_(12)_ = 5.133, *p* < 0.001, Cohen’s d = 22.09).

### Kinematic data

#### Absolute Synchronization Error

**Fig. 4:**
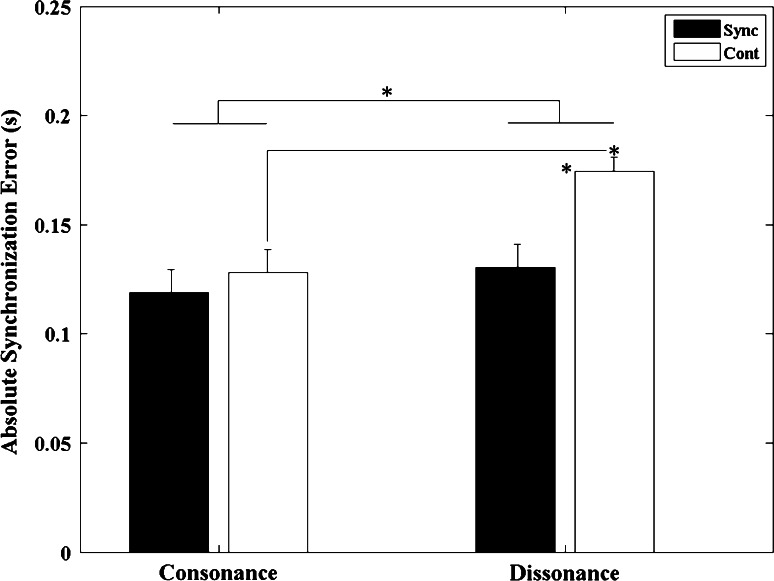
Absolute synchronization error means averaged across all 13 participants for both sound conditions (consonant and dissonant) in the two different stimuli presentation conditions (synchronization and continuation). *Error bars* denote standard errors. Significant comparisons between conditions are highlighted using an *asterisk* (*p < 0.05)

We found a significant main effect for *sounds* (*F*_1, 12_ = 23.397, *p* < 0.001, *η* ^2^ = 0.661) with the absolute synchronization errors for dissonant sounds being significantly larger when compared to consonant sounds. This indicates that performance at matching the specified timing was superior for the consonant compared with the dissonant metronome. Moreover, we found a significant main effect for *task phase* (*F*_1, 12_ = 6.037, *p* = 0.03, *η* ^2^ = 0.335), where again the absolute synchronization errors were significantly larger for the continuation compared with synchronization movements. The interaction between *sounds* and *task* *phase* was also significant (*F*_1, 12_ = 15.716, *p* = 0.002, *η* ^2^ = 0.567). The *t* test revealed that for the dissonant sounds the absolute synchronization errors were greater during the continuation conditions compared with the synchronization conditions (*p* = 0.007) with errors in the continuation dissonant condition being greater than the consonant one (*p* < 0.001) (see Fig. 4).

#### Spread of error

An analysis of the spread of errors showed a significant main effect for *sounds* (*F*_1, 12_ = 43.441, *p* < 0.001, *η* ^2^ = 0.784). The timing variability, as measured by the spread of errors, was significantly greater for dissonant compared with consonant sounds. A significant main effect of *task phase* was also found (*F*_1, 12_ = 10.503, *p* = 0.007, *η* ^2^ = 0.467) where the spread of error was significantly larger for continuation compared with synchronization phases.

**Fig. 5:**
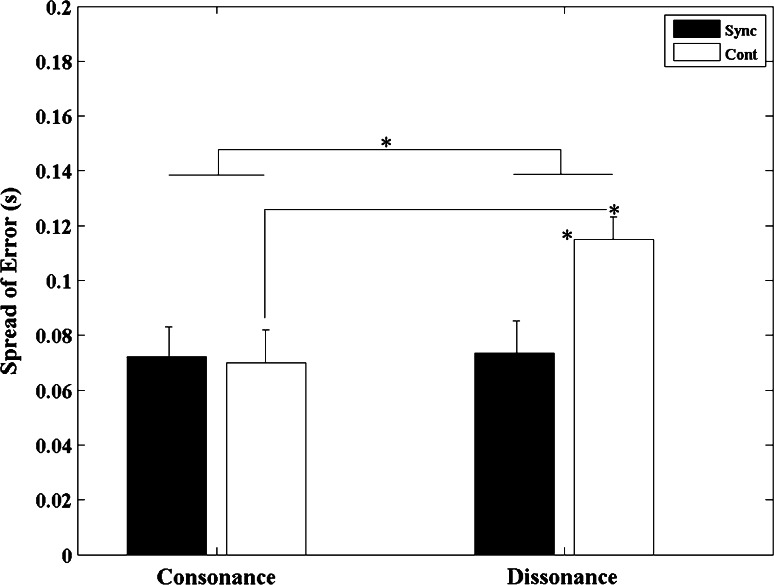
Spread of error averaged across all 13 participants for both consonant and dissonant conditions in the two different stimuli presentation conditions (synchronization and continuation). *Error bars* denote standard errors. Significant comparisons between conditions are highlighted with an *asterisk* (*p < 0.05)

The interaction between *sounds* and *task phase* was also significant (*F*_1, 12_ = 85.452, *p* < 0.001, *η* ^2^ = 0.877). The *t* test revealed that for consonant and also dissonant intervals the spread of error was significantly larger during the continuation compared with the synchronization conditions (*p* < 0.001). During the continuation movements, the spread of error was significantly greater for dissonant compared with consonant sounds (*p* < 0.001) (see Fig. 5).

#### Circularity index

To understand whether the synchronization movements with consonant and dissonant intervals gave rise to different movement trajectory forms, we carried out an analysis on movement harmonicity. Movement harmonicity can be quantified through a circularity index, which is the RMSE between the normalized velocity profile and the perfect harmonic (sinusoidal) motion (semicircle with blue dots in Fig. 7b) and then subtracted from one. Therefore, a perfect circular motion yields a circularity index of one. Discrepancies in the degree of harmonicity for different conditions (consonant/dissonant) would reveal that the dynamics underlying the movement are influenced by the structure of the sound stimuli.

**Fig. 6:**
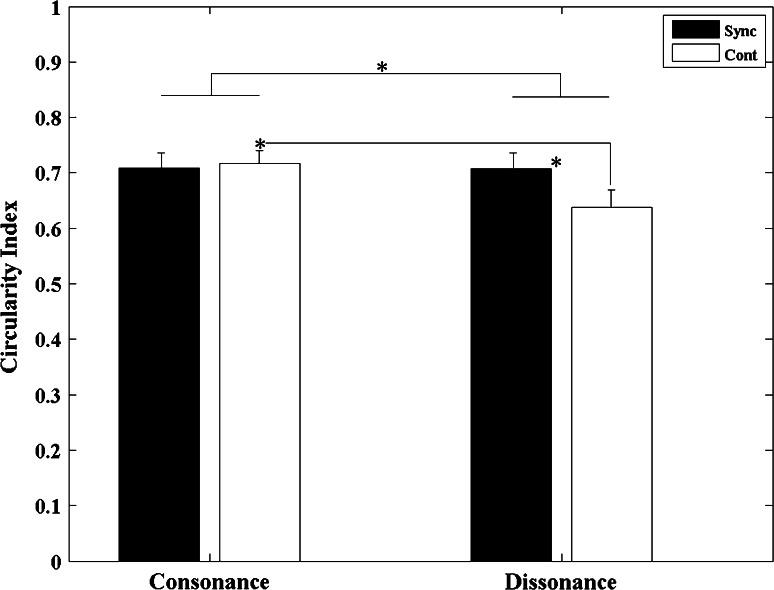
Circularity index averaged across all 13 participants for both consonant and dissonant conditions in the two different stimuli presentation conditions (synchronization and continuation). *Error bars* denote standard errors. Significant comparisons between conditions are highlighted with an *asterisk* (*p < 0.05)

**Fig. 7:**
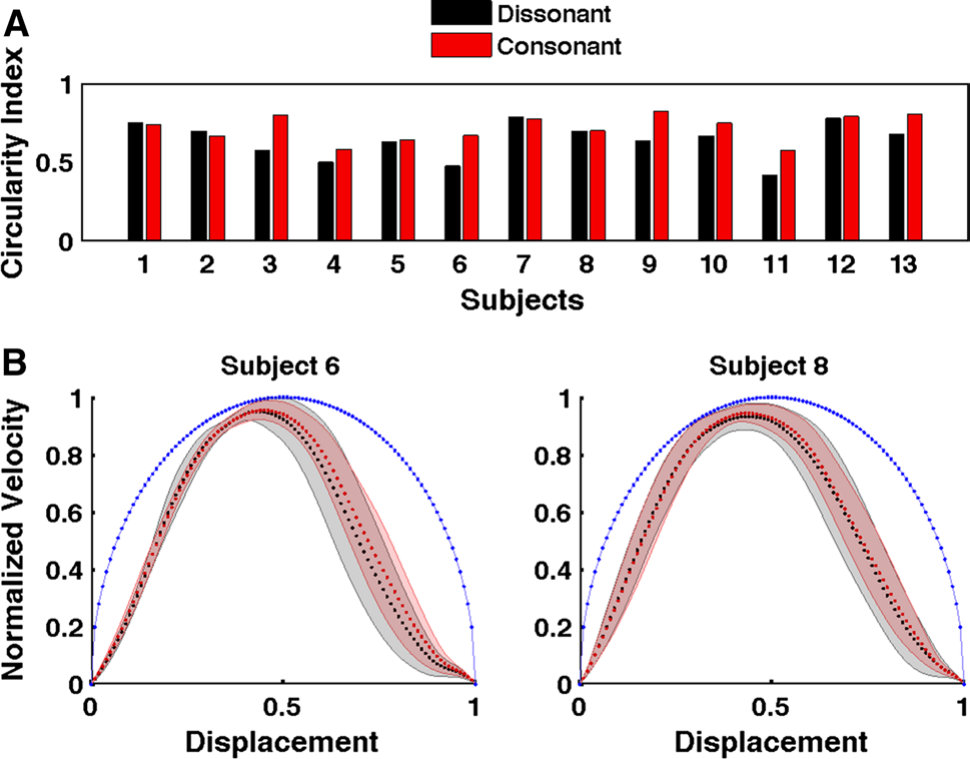
**a** Average circularity index for all 13 participants for both consonant and dissonant conditions during the continuation phase. **b** Averaged data from two participants (6 and 8) when moving with consonant and dissonant metronomes during the continuation phase. The averaged normalized velocity profile plotted against normalized displacement, and shaded regions around the velocity profiles represent *error bars* (SEM). For subject number 6, movements are more circular in form for consonant (*red dots*) than dissonant intervals (*black dots*), while subject number 8 showed a similar pattern of movement circularity for both intervals. The *blue dots* indicate the velocity profile of a perfect sinusoidal movement (color figure online)

The results revealed a significant main effect for *sound* (*F*_1, 12_ = 9.419, *p* = 0.01, *η* ^2^ = 0.44). Furthermore, it was noted that the dynamics of the movements to consonant sounds were significantly more harmonic (larger circularity index) than movements to dissonant sounds. The main effect of *task* phase was also significant (*F*_1, 12_ = 5.433, *p* = 0.038, *η* ^2^ = 0.312) with movements being found to be more circular during the synchronization compared with the continuation phases. The interaction between *sounds* and *task phase* was also found to be significant (*F*_1, 12_ = 10.392, *p* = 0.007, *η* ^2^ = 0.464). The *t* test revealed that for the dissonant intervals the harmonicity of movement was significantly greater (larger circularity index) during the synchronization compared with the continuation phases (*p* = 0.015). Moreover, when moving in the continuation phase to the memory of the metronome, the harmonicity of movements was found to be greater (the circularity index was larger) with consonant compared with dissonant sounds (*p* = 0.008) (see Figs. 6, 7).

#### Tau-G coupling

To understand how the type of information presented through the stimuli might be affecting the subsequent movement, we carried out an information–movement analysis using the tau-coupling model. The intrinsic tau-guide is a mathematical description of how the time to the next beat could be represented by neural structures (Craig et al. 2005). The form of the guide is prospective in nature allowing for the regulation of action. This part of the analysis allows us to see whether the type of information presented (consonant/dissonant) affects the neural representation of the time between beats and the subsequent resonance of that interval.

**Fig. 8:**
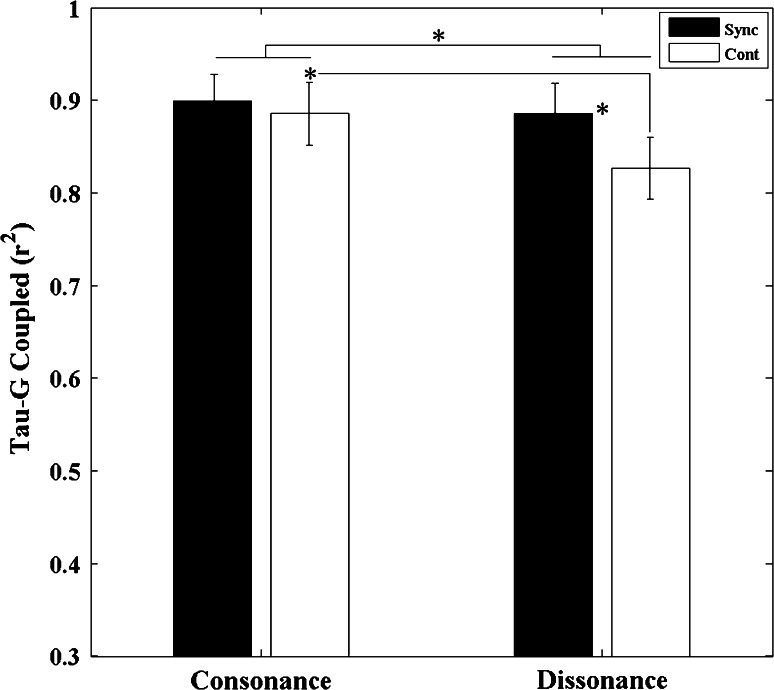
*R* ^2^ values from the tau-coupling regression analysis were averaged across all 13 participants for both consonant and dissonant conditions in the two different stimuli presentation conditions (synchronization and continuation). *Error bars* denote standard errors. Significant comparisons between conditions are indicated with an *asterisk* (*p < 0.05)

To test this, we examined the extent of the coupling between the movement and the information (the intrinsic tau-guide) (see Fig. 3). We measured the strength of coupling (*r*-squared values) when the tau of the movement was plotted against the intrinsic tau-guide (the neural information specifying the time to the next beat). *R* ^2^ values from the tau-coupling regression analysis were calculated for each movement and then averaged for each condition and participant. A significant main effect for *sound* was found (*F*_1, 12_ = 7.666, *p*= 0.017, *η* ^2^ = 0.39) with the *r*-squared values for the tau coupling being significantly greater for the consonant sounds compared with the dissonant sounds. In addition, we found a significant main effect for *task phase* (*F*_1, 12_ = 8.151, *p* = 0.014, *η* ^2^ = 0.404) with tau-coupling *r*-squared values being significantly higher during the synchronization compared with the continuation phases. Moreover, the interaction between *sounds* and *task phase* was also significant (*F*_1, 12_ = 9.151, *p* = 0.011, *η* ^2^ = 0.433). The *t* test revealed that for the dissonant intervals the degree of tau-G coupling was significantly stronger during the synchronization condition as compared to the continuation phase (*p* = 0.005). Moreover, during the continuation phase, the extent of the coupling between the movements and the guide was significantly greater for consonant compared with dissonant sounds (*p* = 0.013) (see Fig. 8).

## Discussion^1^

In this study, we showed that the degree of consonance of the sound presented influenced the types of movement produced after the sound stimulus was removed and the participant continued moving between the two target zones at the same tempo, despite the absence of a metronome. The movement performance measured after exposure to a consonant as compared to a dissonant metronome was found to be less variable and more precise, with a higher percentage of information/movement coupling (tau coupling) and a higher degree of movement circularity (indicating a smoother oscillatory motion). This result suggests that the internal neural resonance of the sound just heard is more accurate when the sound is consonant than when it is dissonant, resulting in better guidance of the movement, which gives rise to more stable movement patterns. If this is the case, then an internal clock model such as the Wing and Kristofferson model (1973) should also consider the multiple aspects present in the structure of auditory cues (e.g., consonant/dissonant pitch intervals). It is worth noting that, in the synchronization phase, when participants were moving under the continual guidance of a metronome, no difference between consonant or dissonant sounds was present either for accuracy or for variability. These results suggest that the continual metronome beat leads to the production of a metric pattern of movement that is independent of the harmonic content of the sounds.

The consonant and dissonant intervals also had an effect on movement harmonicity, with consonant intervals resulting in more sinusoidal movements compared with dissonant ones, with this again being more evident during the continuation phase. Rodger and Craig (2011) showed already that the dynamics of synchronizing movements with continuous sounds were more circular when compared to discrete sounds. Here, our results reinforce the idea that the degree of consonance of sounds influences the shape of oscillatory movements between target zones even during un-paced movement. This result highlights how the level of consonance of the inter-beat intervals plays an important role in governing the pattern of movement even when the auditory guide is no longer present. This suggests that when moving with consonant and dissonant time intervals, the neural structures representing the demarcation of time resonate internally in different ways.

By testing the tau-coupling theory, we found that presenting dissonant intervals leads to a marked decline in the percentage of information/movement coupling. According to Craig et al. (2005), when movements need to be synchronized with acoustic beats, the sensorimotor control of this process involves coupling the tau of the movement (the time to closure of the spatial gap at its current closure rate) onto a tau-guide (a dynamic temporal imprint of the inter-beat interval generated in the brain that continually specifies the time remaining until the next beat will sound). Based on this idea, the dynamic temporal imprint produced when listening to consonant intervals leads to a more robust temporal representation of that time interval. Having a more robust guide would allow for better action control and lead to better synchronization compared with dissonant beats. Craig et al. (2005) also demonstrated that at certain inter-beat intervals (2.5/3 s) there was a decline in the proportion of coupling between prospective information (tau-guide) and hand movements, which resulted in a significant reduction in interceptive performance. Here, we showed that in addition to temporal information specifying the time gap between auditory beats, the context of the auditory information (i.e., the level of consonance of the intervals) also provides information that can enhance the synchronization of movement.

So why does the level of consonance of musical intervals invite different movement strategies during continuation and synchronization tasks? Firstly, it is important to recall that the differences found for consonant over dissonant sounds were particularly emphasized during the continuation phase, suggesting that the quality of a sound will affect the structure of the internal dynamic temporal imprint that guides action when external stimuli are absent. A possible explanation is that during the synchronization task, the stimuli duration can be repeatedly encoded when the metronome is present, allowing for a more precise reproduction of that interval duration. On the other hand, during the continuation phase, participants need to represent and reproduce the metrical pattern from memory. We hypothesized that this is due to different emotional states evoked by the sounds (as shown by the behavioral result), which in turn affects the types of movement produced when external stimuli are absent and subjects continued to move at the same rate from memory (continuation phase). Moreover, it might be due to the diverse feelings of “tension” and “resolution” in dissonant and consonant musical intervals. The concept is well known in music theory: Dissonant intervals increase tension and often lead to a resolution to consonant intervals, which change the primary sensation of tension to a more stable feeling (for a review see Koelsch 2014; Lehne et al. 2013, 2014; Farbood 2012; Sorce 1995). Thus, moving under unresolved (incomplete) auditory events could lead to relatively poor timing performance during the continuation phase. Another reason might be that the perception of the duration of the inter-beat interval evoked by the auditory events may be different (i.e., a disruption of the perception of time is caused by the unpleasant beating in dissonant sounds). Interestingly, it has been shown that emotional valence of music modulates time perception (Droit-Volet et al. 2013). However, further experiments must be carried out to gain a better understanding of the effect of consonance and dissonance intervals on time perception. Either way, we show that the type of sound appears to affect the sensorimotor response, even though the interval duration remains the same.

The hierarchical rating of consonance (i.e., “pleasantness”) and their parallel usage in music composition (Krumhansl 1990) might explain why the degrees of musical tonality affect movement time and trajectory differently in a sensorimotor continuation task. Neuroimaging studies have revealed robust differences in the processing of musical intervals at both cortical (e.g., premotor cortex: Minati et al. 2009) and subcortical levels (e.g., brainstem: Bidelman and Krishnan 2009, 2011), which would imply the involvement of networks involved in both sensory and cognitive processing. A recent review paper has extensively discussed the effects of consonant/dissonant sounds on motor processes in the brain (Koelsch 2014). Moreover, it has been suggested that the preferential encoding of consonant pitch intervals might be rooted in a more robust and coherent neuronal synchronization when compared to dissonant pitch intervals (Tramo et al. 2001; McKinney et al. 2001; Fishman et al. 2001). Importantly, Tierney and Kraus (2013) provided evidence for a link between the ability to synchronize movements to an auditory beat and the consistency of auditory brainstem timing. Thus, a more robust and synchronous phase-locking response in the brainstem when presented with consonant rather than dissonant pitch intervals (Bidelman and Krishnan 2009, 2011) could explain the higher degree of consistency found in this study when subjects synchronize movements to consonant stimuli.

Further evidence suggests that both the cerebellum and the basal ganglia are the cornerstone of an internal timing system (Ivry 1997; Diedrichsen et al. 2003). Recently, Claassen et al. (2013) tested cerebellar disorders (CD) and PD, using a synchronization–continuation paradigm, to decipher the role of the cerebellum and basal ganglia in motor timing. They found that CD participants were less accurate than PD patients during the continuation phase, suggesting a specialized role for the cerebellum in internal timing (Claassen et al. 2013). Hence, it is possible to speculate that consonant pitch intervals may activate the cerebellum more than dissonant ones, and this may account for the better and more precise clocking of fine movements. For a better understanding of this mechanism, it would be interesting to investigate how the sensorimotor system in cooperation with the auditory system extracts relevant information embedded in the musical pitch intervals to control movements in a synchronization–continuation task.

By knowing better why consonant musical pitch intervals can benefit the synchronization of movement compared with their dissonant counterparts, we might be able to use them as auditory guides to improve movement performance in patients with sensory–motor deficits, such as in PD (Rodger et al. 2013). It has been shown that acoustic guides for movement are beneficial in reducing spatial and temporal gait variability in PD patients (Young et al. 2014; Bieńkiewicz et al. 2014; Young et al. 2013). Moreover, the notion that different musical chords evoke different emotions, which in turn can potentially drive the generation of different movement patterns, might be applied to the models of affective engagements with music involving body movement and dance. Further experimental exploration on the relationship between sensorimotor coupling with music and emotion might shed light on why some dances are set to certain kinds of music. It should be noted that the present experiment assessed the perceptual motor ability in a normal population and will be used in the future as a model for testing expert musicians. A tentative hypothesis can be advanced where one might expect that expert musicians will not differ in their performance when synchronizing their movement to consonant and dissonant sound intervals. This putative result would add to our knowledge of the perceptual–motor changes that result from learning a musical instrument.

## Conclusions

In the present study, we tested the effects of musical consonance/dissonance on sensorimotor timing in a synchronization–continuation paradigm during which participants performed reciprocal aiming movements. Remarkably, the analysis of the participants’ movement in the continuation phase revealed that after listening to consonant as opposed to dissonant intervals smaller absolute synchronization errors and spread of errors were found. Furthermore, a higher percentage of movement was tau-coupled and a higher degree of movement circularity was also found. It might be argued that musical pitch combinations caused alterations in perceived tempo during the synchronization phase that, in turn, resulted in a different regulation of motor commands during the continuation phase. Overall, it was found that the harmonic aspects of the musical structure systematically affected both the movement form and timing. We believe that this research yields new insights into the nature of the innate bias that makes consonance perceptually more attractive than dissonance.

## Footnotes

1 Please note that the significant main effects found in the above statistics are not meaningful in light of the significant interactions. For example, while the main effect of sound (*consonant* and *dissonant*) and task phase (*synchronization* and *continuation*) were found to be significant, the interactions between them indicate where these differences are coming from. This is why we mainly focus our discussion around the significant interactions.
